# The Morphology and Morphometric Analysis of the Radius Bone: A Study on Freshly Frozen Cadavers in the Indian Population

**DOI:** 10.7759/cureus.41170

**Published:** 2023-06-30

**Authors:** Sandeep Reddy, Darshan Jain, Karthik Pradyumna, Prajwal R

**Affiliations:** 1 Orthopaedics, Ramaiah Medical College, Bangalore, IND; 2 Orthopaedics, Apollo Hospitals, Bangalore, IND; 3 Orthopaedics, Aster CMI Hospital, Bangalore, IND

**Keywords:** upper extremity, radius fractures, cadaver, elbow prosthesis, elbow joint

## Abstract

Introduction

The radial bone and the radioulnar joint are vital for the physiological and physical stability of the elbow. The prostheses and plates used in cases of radius fracture are designed based on the morphology of the Western population. This could result in a bone-implant mismatch when applied to the Indian population, resulting in complications. Hence, the study aimed to record the normal values of radius morphology in the Indian population.

Methods

A total of 30 (eight male and seven female) freshly frozen cadaveric bilateral upper limbs were chosen. Cadavers with previous surgical scars, deformities, and congenital defects of the upper limb were excluded. The radius was excised, and morphometric parameters were measured with a non-elastic measuring tape and a digital caliper and recorded using GeoGebra software.

Results

All measuring parameters exhibited no significant difference between the right and left side of the bone (p > 0.05), whereas the difference between males and females for most parameters was statistically significant (p < 0.05). The mean difference between the anteroposterior (AP) diameter and transverse diameter of the radial head for the study sample was 0.89 ± 0.06 mm. Thus, the AP diameter was 4% greater than the transverse diameter. The head of the radius was observed to be almost round. The degree of extent of the safe zone was 124.64°, with an average safe arc length of 3.27 ± 0.55 cm.

Conclusion

The morphometric measurements of the radius in the Indian population are different from the Western population.

## Introduction

Around 1.5% of all visits to the emergency room are due to hand and wrist fractures, of which 44% are radial and ulnar fractures [1]. Fragility fracture of the distal radius is common, with a lifetime risk of 15% in women, and is the most common first fracture in the postmenopausal period [2]. Distal radius fracture is the commonest fracture seen in children, accounting for 22.4% of all fractures [3].

The relationship between the radius and ulna is critical for the pronation and supination of the forearm. Hence, the radioulnar joint is called a “functional joint.” A malunited fracture can impair the joint and its movements. It is crucial to re-establish the length, alignment, and rotation of both joints of the forearm to maintain its dynamic function [4,5]. Radial head preservation either by operative fixation or by prosthetic replacement is recommended due to the vital role of the radial head in stabilizing the forearm and elbow. However, most of the radial head prostheses are not based on data studying the Indian population [6]. There exists a mismatch between commercially available prostheses and the required morphological dimensions, leading to radial shortening, painful motion, periarticular ossification, synovitis, and osteoporosis of the capitulum [7]. Despite the benefits of internal fixation of radial fractures, complications arising because of implant-bone mismatch and improper positioning of the pre-contoured plates, such as extensor and flexor tendon complications or malunion, can be avoided by acquiring a thorough understanding of the normal radius morphology [8]. Thus, the study objective was to determine the normal radius morphology in the Indian population and thereby achieve better anatomical alignment in radius fractures after surgery.

## Materials and methods

The present study was a cross-sectional study conducted on 30 radius bones from 15 fresh cadavers (eight males and seven females) of Indian origin at the Department of Orthopedics in a tertiary care hospital. The Institutional Ethics Committee approved the study (ethics committee number: STD-1/EC/060/2015). Informed consent was obtained from the patients or waived. Cadavers with a surgical scar along the forearm, arthritic changes in the elbow and wrist joint, radial fracture, and any congenital anomaly were excluded from the study. In the study conducted by King et al. (2001) [9], the mean difference between the maximum and minimum radial head diameter was 1.7 ± 0.7 mm. With an absolute precision of 0.25 mm and a confidence interval of 95%, the minimum sample size required for the present study was estimated to be 30 radial bones from cadavers. The mean difference between the left and right sides was 0.97 mm (from the pilot study). The α error and power set were 5% and 80%, respectively. The estimated size was 15 on each side; hence, a total of 30 adult radial bones (15 right and 15 left) were studied.

With the elbow flexed and forearm pronated, a posterolateral approach (Kocher approach) was adopted, and the radial head and annular ligament were exposed. The annular ligament was incised from its ulnar distal attachment and elevated. A non-elastic measuring tape was used to measure the length of the annular ligament and safe arc concerning pronation and supination of the forearm. The incision was further extended dorsally along the posterolateral plane (Thompson approach), the part of radial bone between the extensor digitorum communis and extensor carpi radialis brevis muscle was exposed and excised by sharp dissection, and care was taken to divide all attached soft tissues while maintaining the structure and articular surface.

**Figure 1:**
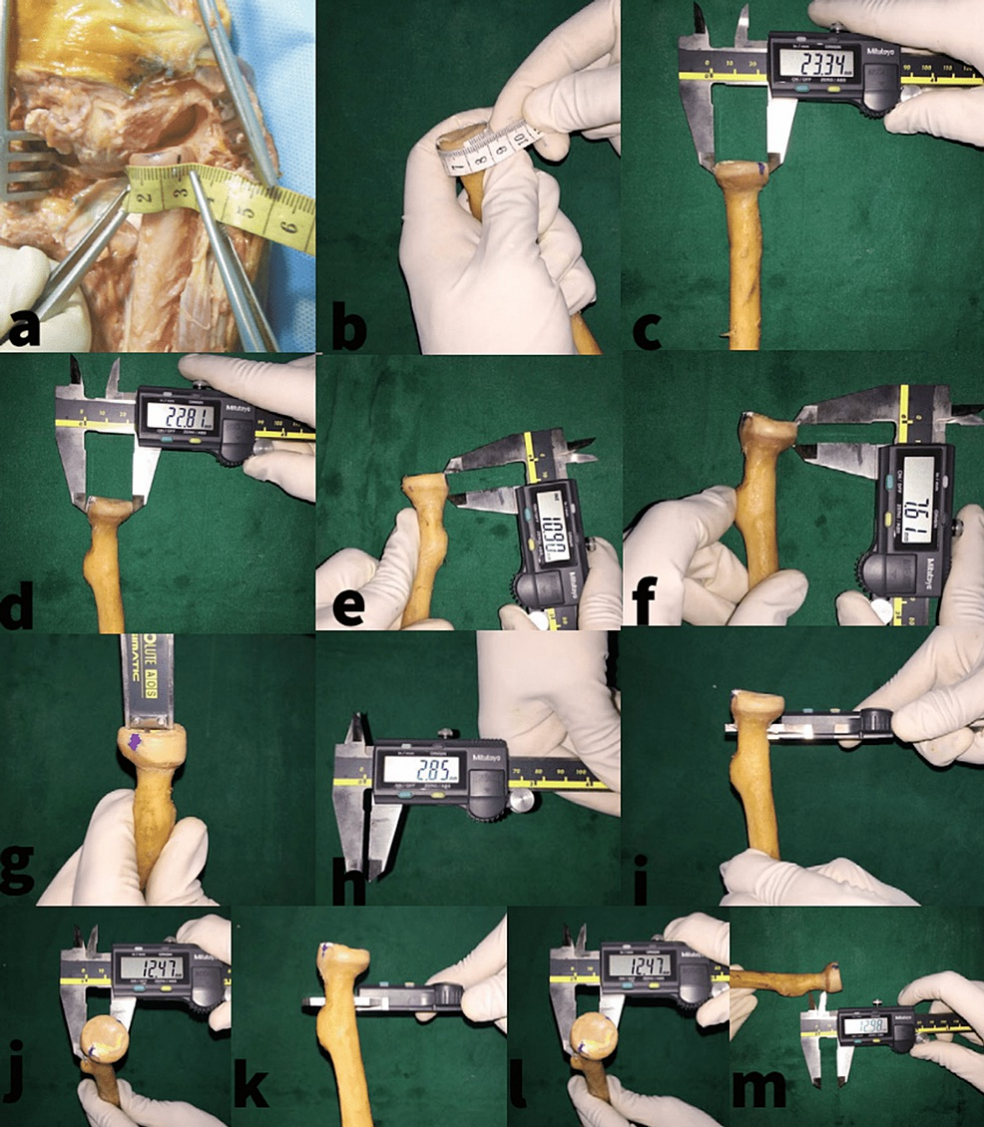
Measurement of the proximal radius a: Measurement of the non-articular surface of the radial head (safe arc) length. b: Measurement of the circumference of the radial head with measuring tape. c: AP measurement of the radial head with a vernier caliper. d: Transverse diameter measurement of the radial head with a vernier caliper. e and f: Medial and lateral radial head height measured with a vernier caliper. g and h: Measurement of articular depth of fovea capitulum radii with depth measure of vernier caliper. i and j: Measurement of the proximal radial neck diameter with a vernier caliper. k and l: Measurement of the distal radial neck diameter with a vernier caliper. m: Measurement of the radial neck length with inner jaws of a vernier caliper. AP: anteroposterior

**Figure 2:**
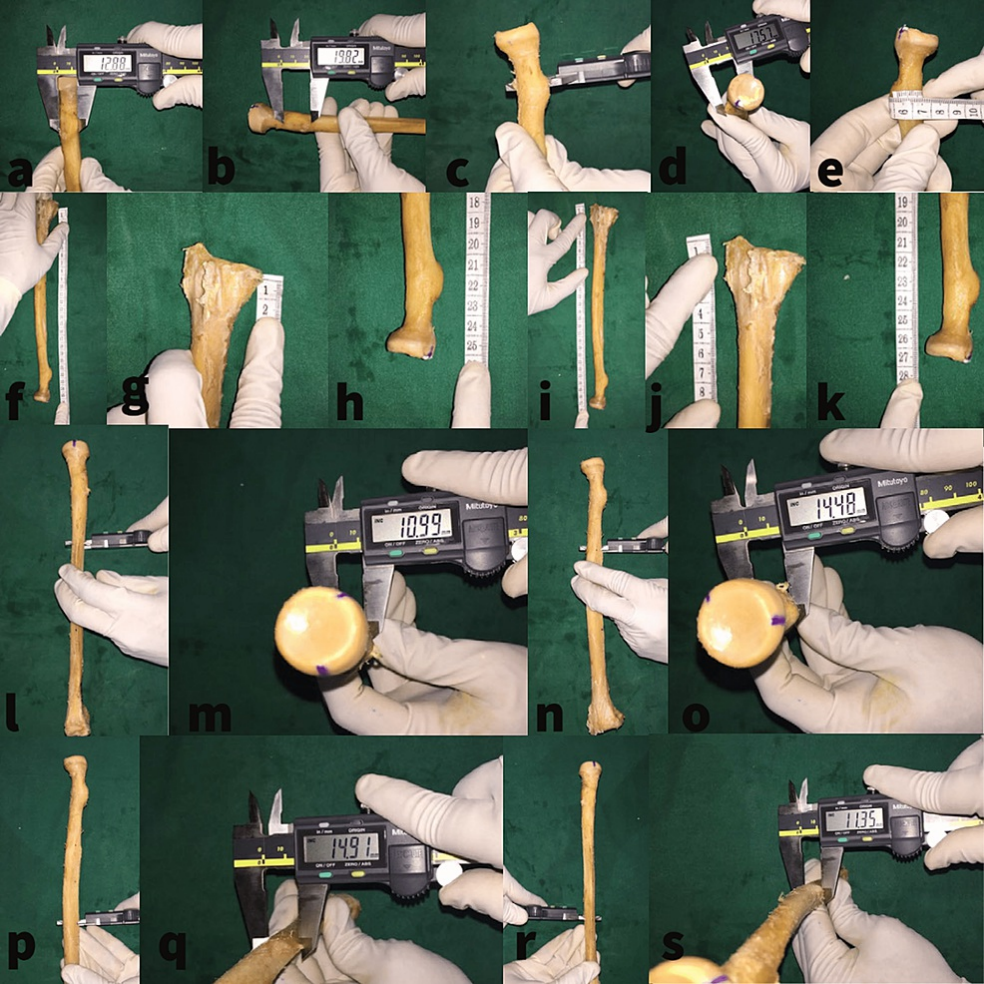
Measurement of the radial shaft a: Measurement of the width of the BT with a vernier caliper. b: Measurement of the BT height with the outer jaw with a vernier caliper. c-e: Measurement of the maximum diameter at the BT with the outer jaws of a vernier caliper. f-h: Length of the radius on the medial side. i-k: Length of the radius on the lateral side. l-s: Measuring the minimum and maximum diameter of the radial shaft at the proximal and distal third junction. BT: bicipital tuberosity

**Figure 3:**
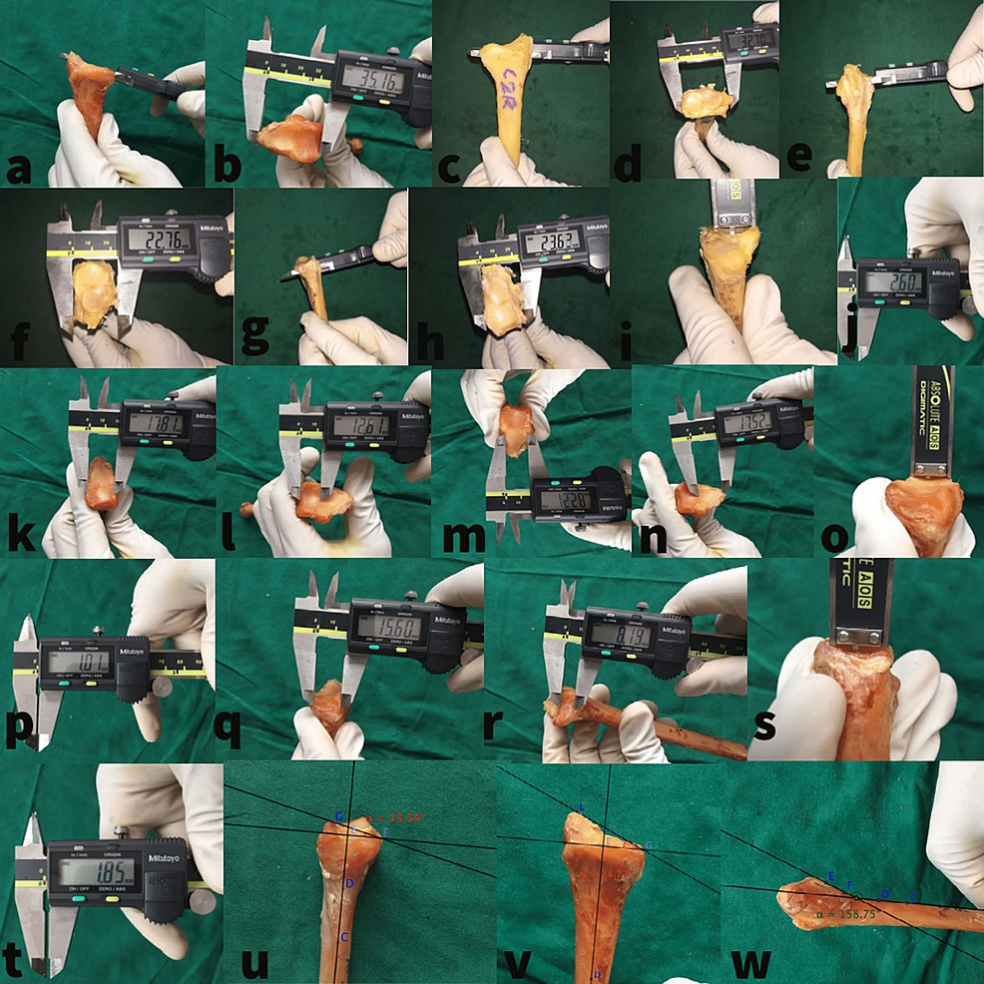
Measurement of the distal radius a and b: Measuring the transverse diameter along the watershed line of the distal radius with a vernier caliper. c and d: Measuring the transverse diameter of the distal radius proximal to the DRUJ. e and f: Measuring the AP diameter of the distal radius along the subchondral surface. g and h: Measuring the AP diameter of the distal radius proximal to the DRUJ. i-l: Measuring the articular surface of the lunate with respect to the maximum AP, transverse diameter, and articular depth. m-p: Measuring the articular surface of the scaphoid with respect to the maximum AP, transverse diameter, and articular depth. q-t: Measuring the articular surface of the ulnar fossa with respect to the maximum AP, superior-inferior diameter, and articular depth. u: Measuring the palmar tilt. v: Measuring the radial inclination. w: Measuring the volar cortical angle. DRUJ: distal radioulnar joint

Morphometric measurements were divided into the proximal radius, radial shaft, and distal radius (Figures 1-3). The circumference of the radial head, anteroposterior (AP) diameter, transverse diameter, medial height, and lateral height were measured. Next, the proximal and distal length as well as the diameter of the radial neck, head-neck angle along the coronal plane, and articular depth of the fovea capitulum radii were measured. The length of the radial shaft at the radial and ulnar side and the maximum and minimum diameter at the proximal and distal third of the radial shaft were measured. The bicipital tuberosity width, length, circumference, and maximum diameter were measured. The height of the styloid process, transverse distal radius diameter along the watershed line, transverse distal radius diameter proximal to the distal radioulnar joint, AP diameter of distal radius along the subchondral plane, AP diameter of distal radius proximal to the distal radioulnar joint, palmar tilt, radial inclination, volar cortical angle, articular surface of lunate, scaphoid and ulnar fossa, curve range of the radial shaft, neck-diaphysis angle, and maximum and minimum inner diameter of the shaft above and below bicipital tuberosity were measured.

We used an indirect approach to evaluate the safe zone through a preliminary anatomical study of the radial head circumference to the safe arc length during the dissection of the cadaver. All parameters were measured using a Mitutoyo Digimatic vernier caliper of precision ±0.02mm, non-elastic measuring tape, and GeoGebra software.

Statistical analysis

Descriptive and inferential statistical analyses were carried out in the present study. Data analysis was carried out using R version 3.5.3 statistical software. Continuous data are presented as mean ± standard deviation (minimum-maximum), and categorical data are presented as number (%). Student’s t-test (two-tailed, independent) was applied to evaluate the difference between sides and gender on study parameters.

## Results

The difference between sides and gender on proximal radial measurements is presented in Table 1.

**Table 1:** Difference between sides and gender on various parameters of the proximal radius *Significant (p-value: 0.05 < p < 0.10) **Moderately significant (p-value: 0.01 < p < 0.05) ***Strongly significant (p-value < 0.01) AP: anteroposterior

Variables	Side		Total	p-value
	Right	Left		
AP diameter of the radial head (in mm)	22.44 ± 2.23	22.25 ± 2.45	22.34 ± 2.30	0.83
Radioulnar diameter of the radial head (in mm)	21.56 ± 2.21	21.34 ± 2.57	21.45 ± 2.36	0.80
Circumference of the radial head (in cm)	7.16 ± 0.80	7.13 ± 0.86	7.15 ± 0.82	0.93
Medial radial head height (in mm)	11.12 ± 1.06	10.58 ± 1.26	10.85 ± 1.18	0.21
Lateral radial head height (in mm)	8.07 ± 1.18	7.97 ± 1.05	8.02 ± 1.10	0.82
Head-neck angle along a coronal plane (in degrees)	137.65 ± 8.14	137.10 ± 9.44	137.38 ± 8.66	0.87
Proximal radial neck diameter (in mm)	12.89 ± 1.60	12.68 ± 1.47	12.79 ± 1.51	0.72
Distal radial neck diameter (in mm)	12.70 ± 1.67	12.57 ± 1.69	12.63 ± 1.65	0.84
Radial neck length (in mm)	11.52 ± 2.36	11.37 ± 2.71	11.44 ± 2.50	0.88
Articular depth (in mm)	2.29 ± 0.45	2.35 ± 0.51	2.32 ± 0.48	0.72
Safe arc length (in cm)	3.27 ± 0.60	3.28 ± 0.52	3.27 ± 0.55	0.95
Variables	Right side		Total	p-value
	Male	Female		
AP diameter of the radial head (in mm)	24.25 ± 1.05	20.37 ± 1.00	22.44 ± 2.23	<0.001***
Radioulnar diameter of the radial head (in mm)	23.35 ± 0.86	19.52 ± 1.19	21.56 ± 2.21	<0.001***
Circumference of the radial head (in cm)	7.79 ± 0.51	6.44 ± 0.21	7.16 ± 0.80	<0.001***
Medial radial head height (in mm)	11.77 ± 0.83	10.37 ± 0.76	11.12 ± 1.06	<0.01**
Lateral radial head height (in mm)	8.84 ± 0.68	7.19 ± 1.02	8.07 ± 1.18	0.01**
Head-neck angle along a coronal plane (in degrees)	134.10 ± 6.70	141.70 ± 8.13	137.65 ± 8.14	<0.001***
Proximal radial neck diameter (in mm)	14.09 ± 1.02	11.51 ± 0.78	12.89 ± 1.60	<0.001***
Distal radial neck diameter (in mm)	13.85 ± 1.24	11.39 ± 0.95	12.70 ± 1.67	<0.001***
Radial neck length (in mm)	12.43 ± 2.72	10.47 ± 1.41	11.52 ± 2.36	0.057
Articular depth (in mm)	2.44 ± 0.50	2.11 ± 0.34	2.29 ± 0.45	0.475
Safe arc length (in cm)	3.63 ± 0.37	2.86 ± 0.56	3.27 ± 0.60	<0.01**
Variables	Left side		Total	p-value
	Male	Female		
AP diameter of the radial head (in mm)	24.23 ± 1.14	19.99 ± 1.13	22.25 ± 2.45	<0.001***
Radioulnar diameter of the radial head (in mm)	23.41 ± 1.11	18.98 ± 1.32	21.34 ± 2.57	<0.001***
Circumference of the radial head (in cm)	7.83 ± 0.51	6.34 ± 0.28	7.13 ± 0.86	<0.001***
Medial radial head height (in mm)	11.12 ± 1.34	9.96 ± 0.90	10.58 ± 1.26	<0.05*
Lateral radial head height (in mm)	8.67 ± 0.76	7.18 ± 0.70	7.97 ± 1.05	<0.01**
Head-neck angle along a coronal plane (in degrees)	133.42 ± 10.25	141.31 ± 6.82	137.10 ± 9.44	<0.01**
Proximal radial neck diameter (in mm)	13.71 ± 1.14	11.52 ± 0.73	12.68 ± 1.47	0.001**
Distal radial neck diameter (in mm)	13.79 ± 1.35	11.17 ± 0.55	12.57 ± 1.69	<0.001***
Radial neck length (in mm)	12.70 ± 3.03	9.85 ± 1.18	11.37 ± 2.71	<0.001***
Articular depth (in mm)	2.45 ± 0.43	2.24 ± 0.61	2.35 ± 0.51	0.149
Safe arc length (in cm)	3.63 ± 0.29	2.89 ± 0.43	3.28 ± 0.52	<0.01**

The mean AP radial head diameter was 22.34 ± 2.30 mm, whereas the main transverse diameter was 21.45 ± 2.36 mm. The difference between AP diameter and transverse radial head diameter was 0.89 ± 0.06 mm. Thus, the AP diameter was 4% greater than the transverse diameter. No statistically significant difference was observed in the left- and right-side proximal radius measurements. The difference between AP and transverse diameters, circumference, medial and lateral head height, head-neck angle, proximal and distal radial neck diameter, and safe arc length in males and females was statistically significant (p < 0.05), whereas the radial neck length and articular depth were statistically insignificant (p > 0.05) (Table 1). The extent of the safe zone was 124.64°, with an average safe arc length of 3.27 ± 0.55 cm. In males, it was 127.29° with an average safe arc length of 3.63 ± 0.52 cm, and in females, it was 121.55° with an average safe arc length of 2.87 ± 0.85 cm.

The difference between sides and gender on radial shaft measurements is presented in Table 2.

**Table 2:** Difference between sides and gender on various parameters of the radial shaft *Significant (p-value: 0.05 < p < 0.10) **Moderately significant (p-value: 0.01 < p < 0.05) ***Strongly significant (p-value < 0.01) BT: bicipital tuberosity, AP: anteroposterior

Variables	Side		Total	p-value
	Right	Left		
Total length of the radius - radial side (in cm)	24.12 ± 2.28	24.08 ± 2.34	24.10 ± 2.27	0.96
Total length of the radius - ulnar side (in cm)	22.91 ± 2.2	22.93 ± 2.29	22.92 ± 2.21	0.98
Bicipital tuberosity process width (in mm)	11.52 ± 2.09	11.65 ± 1.52	11.58 ± 1.79	0.84
Bicipital tuberosity process height (in mm)	17.53 ± 3.04	17.59 ± 2.12	17.56 ± 2.57	0.96
Bicipital tuberosity process circumference	5.09 ± 0.49	5.19 ± 0.57	5.14 ± 0.52	0.61
Maximum diameter at bicipital tuberosity (in mm)	16.34 ± 1.6	16.33 ± 1.41	16.33 ± 1.48	0.99
Maximum diameter of the radial shaft at the proximal third (in mm)	14.58 ± 1.37	14.38 ± 1.50	14.48 ± 1.41	0.71
Minimum diameter of the radial shaft at the proximal third (in mm)	10.34 ± 1.13	10.60 ± 1.29	10.47 ± 1.20	0.55
Maximum diameter of the radial shaft at the distal third (in mm)	12.70 ± 1.50	12.75 ± 1.83	12.73 ± 1.65	0.94
Minimum diameter of the radial shaft at the distal third (in mm)	10.49 ± 1.42	10.57 ± 1.66	10.53 ± 1.52	0.90
Curve range of the lateral edge (in degrees)	5.70 ± 2.53	5.66 ± 2.51	5.68 ± 2.48	0.97
Intramedullary diameter superior to the BT in the AP plane (in mm)	9.91 ± 1.60	10.10 ± 1.72	10.01 ± 1.64	0.76
Intramedullary diameter superior to the BT in the transverse plane (in mm)	10.21 ± 1.55	10.32 ± 1.64	10.27 ± 1.57	0.86
Intramedullary diameter inferior to the BT in the AP plane (in mm)	8.76 ± 1.20	9.13 ± 1.40	8.94 ± 1.30	0.45
Intramedullary diameter inferior to the BT in the transverse plane (in mm)	8.11 ± 1.18	8.64 ± 1.28	8.37 ± 1.24	0.97
Variables	Right side		Total	p-value
	Male	Female		
Total length of the radius - radial side (in cm)	26.05 ± 0.75	21.91 ± 0.92	24.12 ± 2.28	<0.001***
Total length of the radius - ulnar side (in cm)	24.79 ± 0.61	20.77 ± 0.92	22.91 ± 2.20	<0.001***
Bicipital tuberosity process width (in mm)	12.45 ± 2.26	10.45 ± 1.30	11.52 ± 2.09	0.001**
Bicipital tuberosity process height (in mm)	19.71 ± 2.16	15.05 ± 1.64	17.53 ± 3.04	<0.001***
Bicipital tuberosity process circumference	5.44 ± 0.32	4.69 ± 0.28	5.09 ± 0.49	<0.001***
Maximum diameter at bicipital tuberosity (in mm)	17.39 ± 1.23	15.13 ± 1.02	16.34 ± 1.60	<0.01**
Maximum diameter of the radial shaft at the proximal third (in mm)	15.32 ± 1.45	13.73 ± 0.58	14.58 ± 1.37	<0.001***
Minimum diameter of the radial shaft at the proximal third (in mm)	11.05 ± 0.93	9.53 ± 0.72	10.34 ± 1.13	<0.001***
Maximum diameter of the radial shaft at the distal third (in mm)	13.73 ± 1.33	11.53 ± 0.43	12.70 ± 1.50	<0.001***
Minimum diameter of the radial shaft at the distal third (in mm)	11.63 ± 0.84	9.19 ± 0.45	10.49 ± 1.42	<0.001***
Curve range of the lateral edge (in degrees)	5.96 ± 2.82	5.41 ± 2.33	5.70 ± 2.53	0.1007
Neck-diaphysis angle along a coronal plane (in degree)	13.13 ± 1.27	12.96 ± 0.41	13.05 ± 0.94	0.747
Intramedullary diameter superior to the BT in the AP plane (in mm)	11.20 ± 0.60	8.45 ± 0.92	9.91 ± 1.60	<0.001***
Intramedullary diameter superior to the BT in the transverse plane (in mm)	11.42 ± 0.67	8.83 ± 0.95	10.21 ± 1.55	<0.001***
Intramedullary diameter inferior to the BT in the AP plane (in mm)	9.75 ± 0.49	7.63 ± 0.53	8.76 ± 1.20	<0.001***
Intramedullary diameter inferior to the BT in the transverse plane (in mm)	9.02 ± 0.69	7.07 ± 0.57	8.11 ± 1.18	<0.001***
Variables	Left side		Total	p-value
	Male	Female		
Total length of the radius - radial side (in cm)	26.01 ± 0.77	21.87 ± 1.20	24.08 ± 2.34	<0.001***
Total length of the radius - ulnar side (in cm)	24.84 ± 0.82	20.76 ± 1.03	22.93 ± 2.29	<0.001***
Bicipital tuberosity process width (in mm)	12.36 ± 1.19	10.84 ± 1.51	11.65 ± 1.52	<0.01**
Bicipital tuberosity process height (in mm)	18.47 ± 2.24	16.58 ± 1.54	17.59 ± 2.12	0.092
Bicipital tuberosity process circumference	5.59 ± 0.47	4.73 ± 0.18	5.19 ± 0.57	<0.001***
Maximum diameter at bicipital tuberosity (in mm)	17.30 ± 1.11	15.21 ± 0.68	16.33 ± 1.41	<0.001***
Maximum diameter of the radial shaft at the proximal third (in mm)	15.29 ± 1.37	13.34 ± 0.83	14.38 ± 1.50	<0.001***
Minimum diameter of the radial shaft at the proximal third (in mm)	11.46 ± 1.09	9.63 ± 0.65	10.60 ± 1.29	<0.001***
Maximum diameter of the radial shaft at the distal third (in mm)	14.05 ± 1.42	11.26 ± 0.80	12.75 ± 1.83	<0.001***
Minimum diameter of the radial shaft at the distal third (in mm)	11.88 ± 1.01	9.07 ± 0.59	10.57 ± 1.66	<0.001***
Curve range of the lateral edge (in degrees)	5.97 ± 2.80	5.31 ± 2.31	5.66 ± 2.51	0.85
Neck-diaphysis angle along the coronal plane (in degree)	13.14 ± 1.05	13.13 ± 0.42	13.13 ± 0.80	0.05
Intramedullary diameter superior to the BT in the AP plane (in mm)	11.51 ± 0.64	8.49 ± 0.90	10.10 ± 1.72	<0.001***
Intramedullary diameter superior to the BT in the transverse plane (in mm)	11.58 ± 0.93	8.87 ± 0.81	10.32 ± 1.64	<0.001***
Intramedullary diameter inferior to the BT in the AP plane (in mm)	10.11 ± 1.03	8.00 ± 0.75	9.13 ± 1.40	<0.001***
Intramedullary diameter inferior to the BT in the transverse plane (in mm)	9.62 ± 0.85	7.52 ± 0.50	8.64 ± 1.28	<0.001***

No statistically significant difference was observed in the left and right sides of the proximal radius measurements. The difference between measurements of the radial shaft in males and females except for the curve range of lateral edge and neck-diaphysis angle (p > 0.05) were statistically significant (p < 0.05) (Table 2).

The correlation of the sides and gender of the distal radial measurements is presented in Table 3.

**Table 3:** Difference in various parameters of the distal radius between sides and gender *Significant (p-value: 0.05 < p < 0.10) **Moderately significant (p-value: 0.01 < p < 0.05) ***Strongly significant (p-value < 0.01) DRUJ: distal radioulnar joint, AP: anteroposterior

Variables	Side		Total	p-value
	Right	Left		
Height of the styloid process (in mm)	9.83 ± 1.57	9.93 ± 1.64	9.88 ± 1.58	0.87
Distal radial diameter - maximum radioulnar diameter (along the watershed line) (in mm)	32.72 ± 3.46	32.54 ± 3.61	32.63 ± 3.47	0.89
Distal radial diameter - transverse diameter proximal to the DRUJ (in mm)	30.43 ± 3.51	29.96 ± 3.69	30.20 ± 3.55	0.72
Distal radial diameter - maximum AP length (along the subchondral surface) (in mm)	21.64 ± 2.23	21.03 ± 2.24	21.34 ± 2.22	0.46
Distal radial diameter - AP diameter proximal to the DRUJ (in mm)	22.77 ± 2.92	22.40 ± 2.94	22.58 ± 2.89	0.73
Palmar tilt (in degree)	13.43 ± 2.80	12.63 ± 3.15	13.03 ± 2.96	0.46
Radial inclination (in degree)	22.89 ± 2.79	22.28 ± 3.24	22.58 ± 2.99	0.59
Articular surface of the lunate - maximum AP diameter (in mm)	16.45 ± 1.49	16.40 ± 1.76	16.43 ± 1.60	0.94
Articular surface of the lunate - maximum transverse diameter (in mm)	12.14 ± 1.74	11.92 ± 2.12	12.03 ± 1.91	0.75
Articular surface of the lunate - articular depth (in mm)	2.59 ± 0.39	2.50 ± 0.38	2.54 ± 0.38	0.54
Articular surface of the scaphoid - maximum AP diameter (in mm)	13.43 ± 2.14	13.06 ± 1.73	13.24 ± 1.93	0.61
Articular surface of the scaphoid - maximum transverse diameter (in mm)	16.06 ± 2.17	15.97 ± 1.41	16.02 ± 1.80	0.90
Articular surface of the scaphoid - articular depth (in mm)	1.31 ± 0.48	1.43 ± 0.49	1.37 ± 0.48	0.50
Ulnar fossa articular surface - AP diameter (in mm)	14.67 ± 2.08	14.37 ± 1.18	14.52 ± 1.67	0.63
Ulnar fossa articular surface - transverse diameter (in mm)	7.60 ± 1.23	7.87 ± 0.96	7.74 ± 1.09	0.50
Ulnar fossa articular surface - articular depth (in mm)	1.69 ± 0.51	1.57 ± 0.36	1.63 ± 0.43	0.46
Volar cortical angle (in degree)	29.16 ± 4.85	29.29 ± 4.27	29.22 ± 4.49	0.60
Variables	Gender (right side)		Total	p-value
	Male	Female		
Height of the styloid process (in mm)	10.99 ± 1.04	8.50 ± 0.80	9.83 ± 1.57	<0.001***
Distal radial diameter - maximum radioulnar diameter (along the watershed line) (in mm)	35.31 ± 1.90	29.76 ± 2.13	32.72 ± 3.46	<0.001***
Distal radial diameter - transverse diameter proximal to the DRUJ (in mm)	33.04 ± 2.1	27.45 ± 2.06	30.43 ± 3.51	<0.001***
Distal radial diameter - maximum AP length (along the subchondral surface) (in mm)	23.19 ± 1.36	19.87 ± 1.61	21.64 ± 2.23	<0.001***
Distal radial diameter - AP diameter proximal to the DRUJ (in mm)	24.79 ± 2.27	20.46 ± 1.51	22.77 ± 2.92	<0.001***
Palmar tilt (in degree)	13.88 ± 3.61	12.93 ± 1.60	13.43 ± 2.80	0.84
Radial inclination (in degree)	23.33 ± 2.13	22.39 ± 3.51	22.89 ± 2.79	0.23
Articular surface of the lunate - maximum AP diameter (in mm)	17.52 ± 0.89	15.22 ± 0.99	16.45 ± 1.49	<0.001***
Articular surface of the lunate - maximum transverse diameter (in mm)	13.30 ± 1.36	10.82 ± 1.02	12.14 ± 1.74	<0.001***
Articular surface of the lunate - articular depth (in mm)	2.79 ± 0.34	2.35 ± 0.32	2.59 ± 0.39	0.024*
Articular surface of the scaphoid - maximum AP diameter (in mm)	14.61 ± 2.26	12.07 ± 0.86	13.43 ± 2.14	0.015*
Articular surface of the scaphoid - maximum transverse diameter (in mm)	17.45 ± 1.81	14.47 ± 1.29	16.06 ± 2.17	<0.001***
Articular surface of the scaphoid - articular depth (in mm)	1.54 ± 0.54	1.05 ± 0.21	1.31 ± 0.48	0.05*
Ulnar fossa articular surface - AP diameter (in mm)	16.12 ± 1.32	13.01 ± 1.42	14.67 ± 2.08	0.001**
Ulnar fossa articular surface - transverse diameter (in mm)	8.20 ± 1.06	6.91 ± 1.09	7.60 ± 1.23	<0.05*
Ulnar fossa articular surface - articular depth (in mm)	1.93 ± 0.51	1.42 ± 0.36	1.69 ± 0.51	0.05*
Length of the annular ligament (in cm)	4.25 ± 0.19	3.84 ± 0.11	4.06 ± 0.26	<0.001***
Volar cortical angle (in degree)	29.41 ± 5.12	28.87 ± 4.92	29.16 ± 4.85	0.29
Variables	Gender (left side)		Total	p-value
	Male	Female		
Height of the styloid process (in mm)	11.14 ± 1.25	8.54 ± 0.54	9.93 ± 1.64	<0.001***
Distal radial diameter - maximum radioulnar diameter (along the watershed line) (in mm)	35.36 ± 2.14	29.31 ± 1.51	32.54 ± 3.61	<0.001***
Distal radial diameter - transverse diameter proximal to the DRUJ (in mm)	32.84 ± 1.95	26.67 ± 1.88	29.96 ± 3.69	<0.001***
Distal radial diameter - maximum AP length (along the subchondral surface) (in mm)	22.67 ± 1.52	19.16 ± 1.14	21.03 ± 2.24	<0.001***
Distal radial diameter - AP diameter proximal to the DRUJ (in mm)	24.61 ± 1.90	19.87 ± 1.41	22.40 ± 2.94	<0.001***
Palmar tilt (in degree)	12.64 ± 4.08	12.61 ± 1.94	12.63 ± 3.15	0.99
Radial inclination (in degree)	22.58 ± 3.50	21.94 ± 3.15	22.28 ± 3.24	0.10
Articular surface of the lunate - maximum AP diameter (in mm)	17.70 ± 1.00	14.92 ± 1.11	16.40 ± 1.76	<0.001***
Articular surface of the lunate - maximum transverse diameter (in mm)	13.13 ± 1.96	10.53 ± 1.34	11.92 ± 2.12	0.011**
Articular surface of the lunate - articular depth (in mm)	2.74 ± 0.27	2.22 ± 0.28	2.50 ± 0.38	<0.001***
Articular surface of the scaphoid - maximum AP diameter (in mm)	13.77 ± 1.82	12.24 ± 1.28	13.06 ± 1.73	0.003**
Articular surface of the scaphoid - maximum transverse diameter (in mm)	16.97 ± 0.99	14.84 ± 0.81	15.97 ± 1.41	<0.001***
Articular surface of the scaphoid - articular depth (in mm)	1.72 ± 0.50	1.11 ± 0.18	1.43 ± 0.49	<0.001***
Ulnar fossa articular surface - AP diameter (in mm)	14.95 ± 0.84	13.70 ± 1.21	14.37 ± 1.18	0.011*
Ulnar fossa articular surface - transverse diameter (in mm)	8.35 ± 0.93	7.33 ± 0.71	7.87 ± 0.96	0.001**
Ulnar fossa articular surface - articular depth (in mm)	1.74 ± 0.36	1.38 ± 0.26	1.57 ± 0.36	0.01*
Volar cortical angle (in degree)	29.53 ± 4.79	29.01 ± 3.94	29.29 ± 4.27	0.03*

The proximal radius measurements on the left and right sides did not show any statistically significant differences. With the exception of palmar tilt, radial inclination, and length of the annular ligament (p > 0.05), there was a statistically significant difference between measures of the distal radius in males and females (p < 0.05) (Table 3).

## Discussion

Displaced and comminuted radial bone fractures are treated with internal fixation of plates and screws or reconstruction of the radial head. The knowledge regarding the bone’s accurate size and shape helps in appropriate implant selection to treat the fracture and prevent complications.

Several methods such as computed tomography, radiography, and cadaveric examination can be applied to evaluate the anatomical parameters of the radius bone [6,10,11]. The application of radiography as a tool for evaluating morphometric measurements of the distal radius has been criticized by a few authors who have found that forearm supination increases and pronation decreases the apparent measurements of radial inclination, radial height, and palmar tilt significantly [11,12]. The measurements of the left- and right-side radii were not significantly different in this investigation (p > 0.05). The work by Hollevoet et al. [13] suggested using the healthy contralateral side for morphometric comparison. The cadaveric-based approach is advantageous as it is a direct method of study, and therefore, we opted for the cadaveric approach. The current work provides a basis for designing anatomical prostheses and pre-contoured plates based on scientific principles. Although radial head plates come in a variety of sizes, even anatomically pre-contoured plates can bend due to the limited correlation between some anatomical factors, leading to subpar function [14]. The precise geometry of the radius is still under debate because the proximal radius has a complicated irregular shape. In addition, a study investigating the anatomical fit of six different plates to the radius bone in cadavers observed that in most cases, the tested devices were not an adequate fit [14].

The difference between the transverse and AP radial head diameters in this study was 0.89 ± 0.06 mm. Thus, the AP diameter was 4% greater than the transverse diameter, and the radial head was approximately round. This conclusion was in contrast to the findings of the study by Kuhn et al. [15], which found that the radial head is ovoid with an average fluctuation in diameter of 2.5 mm at the level of the proximal radioulnar joint. Also, we discovered that there was a significant difference between the sexes in the AP and transverse radial head dimensions.

For optimum performance, radial head fractures must be fixed or replaced with a prosthetic. To avoid endangering the proximal radioulnar joint, Giannicola et al. (2012) [16] recommended doing the radial head and neck fracture osteosynthesis in the safe zone. Using an indirect method, we performed a preliminary anatomical study of the radial head circumference to safe arc length during the dissection of the cadaver. We came to the conclusion that the safe zone’s average degree of extent was 124.64°, with an average safe arc length of 3.27 ± 0.55 cm.

In the present study, the mean AP diameter was 22.34 ± 2.30 mm, and the transverse diameter was 21.45 ± 2.36 mm. These measurements were less than those in the study of Beredjiklian et al. [17] published in 1999 and Swieszkowski et al. [18] published in 2001. To better understand the pathophysiology of a biceps tendon rupture and facilitate surgical procedures such as the reconstruction of the biceps tendon and the radial head prosthesis and implantation during the reconstruction of proximal head trauma, it is also crucial to determine the dimensions of the bicipital tuberosity and its angular relationship with the radial head [19].

In the present study, the mean bicipital tuberosity (BT) width, height, and circumference were 11.58 ± 1.79 mm, 17.56 ± 2.57 mm, and 5.14 ± 0.52 mm, respectively, and the maximum diameter of the radial shaft at the level of BT was 16.33 ± 1.48 mm. We observed that the height of the BT was highly variable. This finding was similar to that of Rajasree et al. (2016) [20], where the dimensions were recorded from the same ethnic population.

Today, the majority of Indian orthopedic surgeons use the distal radius morphometric description, as proposed by Gartland et al. in 1951 [21] in the assessment and treatment of distal radius fractures. The mean palmar tilt and radial inclination in the current study were 13.03 ± 2.96 and 22.58 ± 2.99 degrees, respectively. The palmar tilt was lower in the study by Gartland et al. (11°) although the mean radial inclination was the same (23°).

The study puts forward a metric that is gender-specific and centered around the Indian population to produce radial head prostheses and internal fixation plates. The limitations of this study include its limited sample size and that it is a single-centered study. A larger multicentric clinical study to verify the measurements from the present study would further strengthen the accuracy of these readings.

## Conclusions

The present study defines the normal anatomical parameters of the radius in the Indian population through a cadaveric study. The present study confirmed that the morphometric measurements of the radius in the Indian population were not the same as the Western population. Therefore, internal plate size and radial head prostheses need to be compatible with the Indian population to avoid complications due to implant-bone mismatch. Further knowledge about the size and shape is essential while designing the radial head prosthesis and pre-contoured plates to fix fractures and select the right implants. While reconstructing the biceps tendon, measuring bicipital tuberosity and its angular relationship with the radial head is important for a successful surgery.

## References

[1] Nellans KW, Kowalski E, Chung KC (2012). The epidemiology of distal radius fractures. Hand Clin.

[2] Kalia RB, Agarwal AC (2014). Fragility fractures of the distal radius. J Orthop Trauma Rehabilitation.

[3] Tandon T, Shaik M, Modi N (2007). Paediatric trauma epidemiology in an urban scenario in India. J Orthop Surg (Hong Kong).

[4] Morrey BF (2002). General deep approaches to the elbow: posterior approaches. Tech Shoulder Elbow Surg.

[5] Jupiter JB, Kellam JF (2003). Diaphyseal fractures of the forearm. Skeletal trauma: fractures, dislocations and ligamentous injuries, third edition.

[6] Sridhar S, Jayasree N, Srinivas HL, Shankar DK, Chidambaram S (2015). Morphometry of radial head and neck length in the population of Andhra Pradesh. Int Anat Sci.

[7] Morrey BF, Askew L, Chao EY (1981). Silastic prosthetic replacement for the radial head. J Bone Joint Surg Am.

[8] Mathews AL, Chung KC (2015). Management of complications of distal radius fractures. Hand Clin.

[9] King GJ, Zarzour ZD, Patterson SD, Johnson JA (2001). An anthropometric study of the radial head: implications in the design of a prosthesis. J Arthroplasty.

[10] Singh TS, Sadagatullah AN, Yusof AH (2015). Morphology of distal radius curvatures: a CT-based study on the Malaysian Malay population. Singapore Med J.

[11] Pennock AT, Phillips CS, Matzon JL, Daley E (2005). The effects of forearm rotation on three wrist measurements: radial inclination, radial height and palmar tilt. Hand Surg.

[12] Johnson PG, Szabo RM (1993). Angle measurements of the distal radius: a cadaver study. Skeletal Radiol.

[13] Hollevoet N, Van Maele G, Van Seymortier P, Verdonk R (2000). Comparison of palmar tilt, radial inclination and ulnar variance in left and right wrists. J Hand Surg Br.

[14] Ikeda M, Sugiyama K, Kang C, Takagaki T, Oka Y (2005). Comminuted fractures of the radial head. Comparison of resection and internal fixation. J Bone Joint Surg Am.

[15] Kuhn S, Burkhart KJ, Schneider J, Muelbert BK, Hartmann F, Mueller LP, Rommens PM (2012). The anatomy of the proximal radius: implications on fracture implant design. J Shoulder Elbow Surg.

[16] Giannicola G, Manauzzi E, Sacchetti FM, Greco A, Bullitta G, Vestri A, Cinotti G (2012). Anatomical variations of the proximal radius and their effects on osteosynthesis. J Hand Surg Am.

[17] Beredjiklian PK, Nalbantoglu U, Potter HG, Hotchkiss RN (1999). Prosthetic radial head components and proximal radial morphology: a mismatch. J Shoulder Elbow Surg.

[18] Swieszkowski W, Skalski K, Pomianowski S, Kedzior K (2001). The anatomic features of the radial head and their implication for prosthesis design. Clin Biomech (Bristol, Avon).

[19] Mazzocca AD, Cohen M, Berkson E, Nicholson G, Carofino BC, Arciero R, Romeo AA (2007). The anatomy of the bicipital tuberosity and distal biceps tendon. J Shoulder Elbow Surg.

[20] Rajasree G, Sucharitha T, Karuppiah DS, Hema L (2016). Morphology and morphometry of proximal dry radii in south coastal population. IJRDO.

[21] Gartland JJ Jr, Werley CW (1951). Evaluation of healed Colles’ fractures. J Bone Joint Surg Am.

